# A Flexible, Microfluidic, Dispensing System for Screening Drug Combinations

**DOI:** 10.3390/mi11100943

**Published:** 2020-10-18

**Authors:** Mark Davies, Mannthalah Abubaker, Lorraine Bible

**Affiliations:** 1 Bernal Institute, University of Limerick, Limerick V94 T9PX, Ireland; Mannthalah.Abubaker@ul.ie (M.A.); Lorraine.Bible@ul.com (L.B.); 2 Hooke Bio Ltd., Clare V14 XH92, Ireland

**Keywords:** dilutions, Microfluidics, drug combinations, screening, droplets

## Abstract

It is known that in many cases a combination of drugs is more effective than single-drug treatments both for reducing toxicity and increasing efficacy. With the advent of organoid screens, personalised medicine has become possible for many diseases. Automated pipetting to well plates is the pharmaceutical industry standard for drug screening, but this is relatively expensive and slow. Here, a rotary microfluidic system is presented that can test all possible drug combinations at speed with the use of droplets. For large numbers of combinations, it is shown how the experimental scale is reduced by considering drug dilutions and machine learning. As an example, two cases are considered; the first is a three-ring and three radii configuration and the second is a four ring and forty-eight radii configuration. Between these two, all other cases are shown to be possible. The proposed commercial instrument is shown to be flexible, the user choosing which wells to fill and which driver-computational sub-routine to select. The major issues addressed here are the programming theory of the instrument and the reduction of droplets to be generated by drug dilutions and machine learning.

## 1. Introduction

Combinatorial drug therapy has shown promising results for treating complex diseases such as cancer, HIV, or malaria [1,2,3]. The administration of multiple compounds has shown a reduction in side effects and toxicity compared to monotherapy and lowers the risk of resistance development [4,5,6,7,8]. With drug combinations, a more targeted treatment known as personalized medicine becomes possible as different drugs can be tested on the cells of individual patients, thus customizing the care towards their specific response [9,10,11,12,13]. This will be particularly important with the use of 3D cell culture technologies such as organoids. Organoids can be established from patient-derived healthy and tumour tissue samples that self-organise in culture to acquire in-vivo like organ complexity that have been shown to be more translatable to in-vivo physiology [14,15,16]. However, to achieve this, large libraries consisting of many active ingredients must be screened to find the required response [17]. Currently, drug combinatorial tests are limited to combinations of two due to constraints in modern technology specific to drug screening [18]. The cost and time that is required by the existing technology make it unfeasible to test for all possible drug combinations [19,20]. Furthermore, to explore all the possible combinations leads to challenges associated with the ‘Combinatorial Explosion’ in which the number of combinations increases factorially with the number of drugs tested [21].

**Figure 1:**
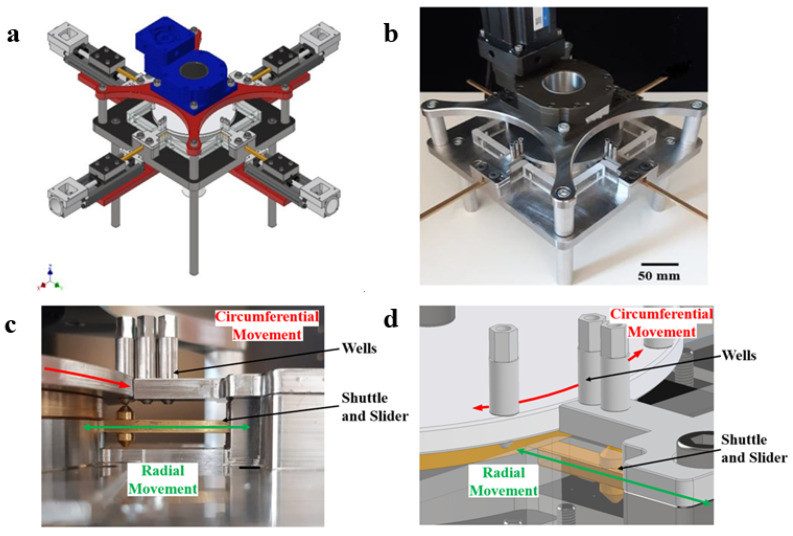
Prototype [22]. (**a**) CAD image of prototype. (**b**) Four-by-two prototype. (**c**) Circumferential movement of the ring is denoted by the red arrow and radial movement of the shuttle by the green arrow. (**d**) Corresponding CAD image.

**Figure 2:**
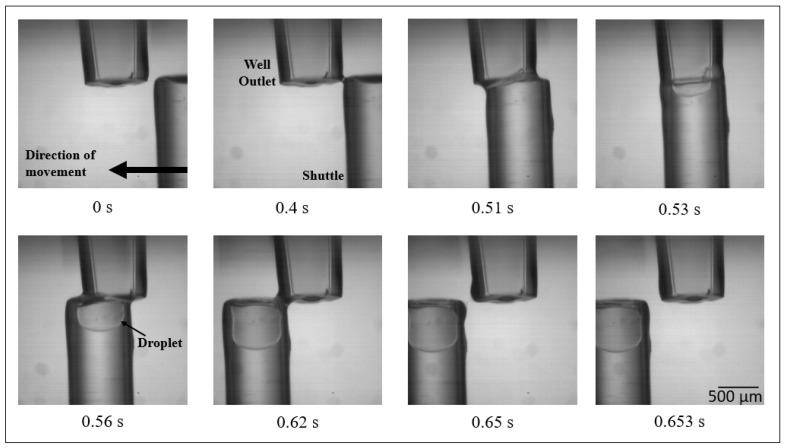
Gap Switch Technology (GST), Droplet Generation and Mixing. High-speed camera images taken of the GST. Images were taken using an IDT X-Stream XS-4 CMOS Camera at 1000 frames per second. The droplets are completely surrounded by silicone oil and therefore never contact a solid surface. This eliminates carry-over contamination and makes the system continuously reusable. Shuttle velocity is approximately 4mm/s. The dispensed droplet can be seen in the lower tube. The tubing is PTFE which attracts the oil and repels the aqueous, hence the wrapped droplets.

Figure 1 shows a prototype for ultra-high throughput, rotary, microfluidic drug screening. A novel method of dispensing aqueous-in-oil droplets, Gap Switch Technology (GST) is described in more detail in [22]. Stainless steel wells are filled from above, droplets are then dispensed from a 0.5 mm internal diameter outlet from below. Wells are designed to contain a range of biological fluids. Interfacial and capillary forces prevent fluid flow between dispensing. When a brass dispensing shuttle aligns with the well outlet, a liquid bridge is formed, and fluid flow is induced. The aqueous phase then encounters the immiscible carrier silicone oil, where the aqueous phase necks and is sheared off as the shuttle moves past the well. The aqueous phase is wrapped in the oil compartmentalising a droplet, Figure 2. This process is repeated to dispense and mix a second droplet. The droplets are efficiently mixed by having them of a different volume, hence the Laplace pressure difference drives mixing, vorticity. The mixed droplets are then transferred into a reusable polytetrafluoroethylene (PTFE) channel, for incubation and analysis.

An industry standard 96 well plate has a total assay volume in the order of 10^−9^ m^3^. The mixed droplet volume is of order 6 × 10^−11^ m^3^, making it significantly smaller than a well. There is no known reason why it should not be smaller. The aim is to provide a faster and lower-cost method of combinatorial drug screening. Operating costs are reduced by removing the reliance on pipettes and well plates and using smaller assay/reagent samples and removing the need for complex robotic set-ups required for current drug screening technologies [23]. Dispensing time is reduced due to the instruments enhanced mixing and encapsulation. These mass transport properties are due to the high surface area to volume ratio, facilitating fast and controlled heating of droplets and creating shorter diffusion distances between molecules [24,25,26,27,28].

The GST has been designed to be scaled into a rotational design, Figure 1, to increase microfluidic throughput. The design allows movement of wells by concentric rotating rings into the pathway of radial shuttle movements. This improves the throughput of the system through parallelization [29]. The shuttle can then selectively dispense and mix drugs from these wells along the radial shuttle pathways. The flexibility of the design is due to the many potential configurations, depending on which wells are filled and which computational sub-routine is selected. Four instrument strategies are discussed:

1. Measure every combination without dilutions.
2. Measure every meaningful combination with dilutions.
3. For large numbers of droplets, measure 1 and 2 with machine learning.
4. For 3 measure single dilutions first, then next dilution until synergy is found.

## 2. Three-by-Three Example

**Figure 3:**
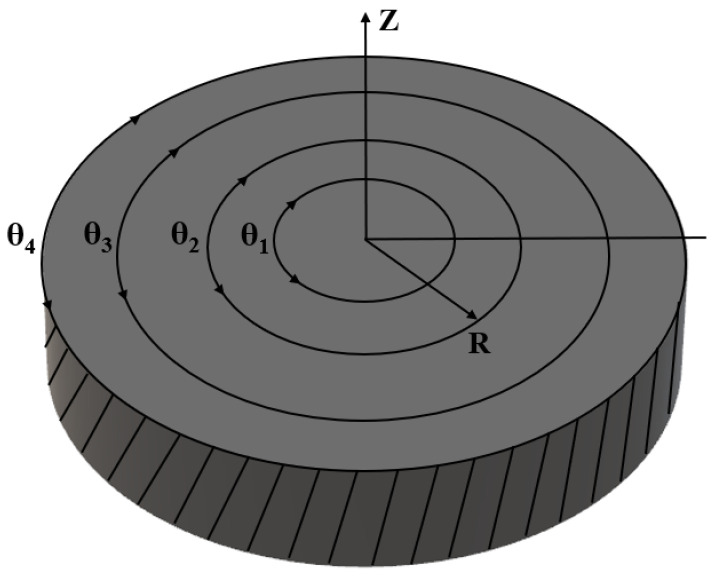
Nomenclature.

**Figure 4:**
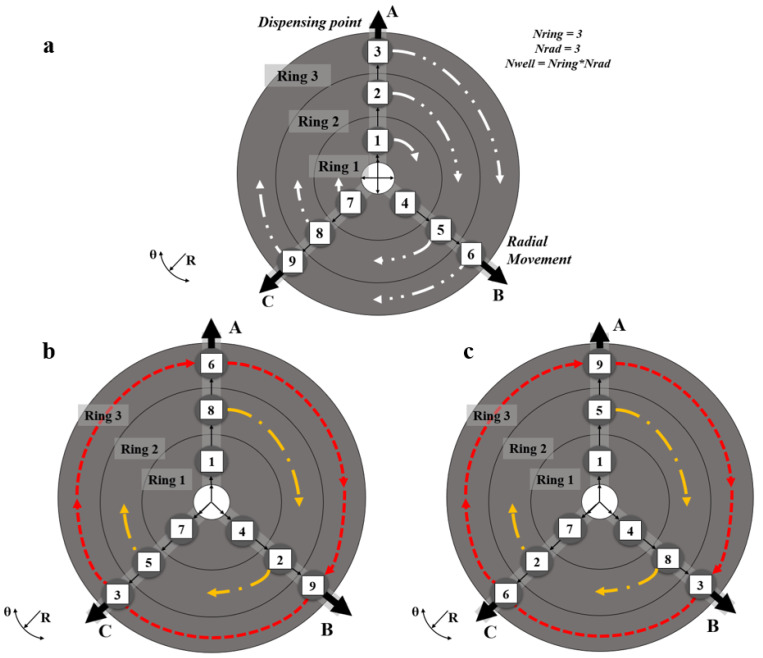
Three-by-Three Configuration. (**a**) Overview of a 3 × 3 configuration. Drug combinations occur through either circumferential movement, the movement of a droplet from a well on one radius to a well on another radius, or through radial movements, the movement of a droplet along the same radius. There is a total of 84 combinations, requiring 28 positions for all combinations. (**b**) shows rotor position after movement. The movement of a droplet on ring 2 from a well on radius 1 to a well on radius 2 (a. 120° rotation) and a droplet on ring 3 moving from a well on radius 1 to a well on radius 3 (a 240° rotation). (**c**) shows the next positions following (**b**). A, B and C are the outlet lines.

To introduce the system a three-by-three configuration is considered, nomenclature is given in Figure 3. Figure 4 shows a three radii, three ring build. It operates through three types of movements;

1. Circumferential movements: movement from one well to another well on the same ring,
2. Radial movements: movement from a well on one ring to a well on a different ring,
3. Mixed movements: a combination of radial and circumferential movements.

There are nine filled wells containing either drugs, cells or organoids. The figure shows how the different wells, rings and radii interact both mechanically and fluid mechanically. The shuttles move radially to collect droplets from wells on the same radius and the rings would move circumferentially to allow the shuttle to collect droplets from wells on different radii. The shuttle can pass under a well without taking a droplet if it is moving at a high enough velocity. If drugs are combined in sets of three, there will be a total of eighty-four combinations, Equation (1). This requires the instrument to take up twenty-eight positions. The mathematics of the instrument is explained in the next section. For commercialisation, a maximum forty-eight by four configuration is proposed. This is twice the number of wells on a ninety-six well plate which is considered the industry standard [30].

## 3. Discrete Analysis

The binomial equation [31],

(1) $${}_{n}C_{r}\;=\frac{\;n!}{r!\left(n-r\right)!}$$

applied to the instrument design, Figure 3 and Figure 4,

(2) $$N_{p}\;N_{\mathrm{rad}}\;=\;\frac{\left(N_{\mathrm{ring}}\;N_{\mathrm{rad}}\right)!}{r!\;\left(N_{\mathrm{ring}}\;N_{\mathrm{rad}}\;-\;r\right)!}$$

describes its operation without dilutions. The number of droplets to complete a test is determined by knowing the number of radii, the configuration and the number of combinations to test. The number of droplets,

(3) $${}_{n}C_{r}\;=\;N_{p}\;N_{\mathrm{rad}}$$

maybe derived in two, depending on how they are formed,

(4) $${}_{n}C_{r}\;=\;{{}_{n}C_{r}}_{r}\;+\;{{}_{n}C_{r}}_{c}$$

If dilutions are used,

(5) Cr′n=Crr′n+Crc′n

in which case,

(6) Crn>Cr′n

which always gives a throughput advantage.

**Figure 5:**
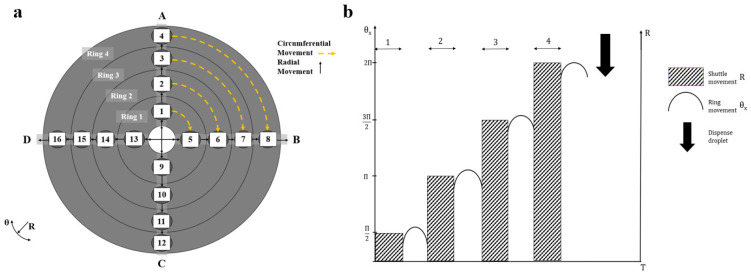
Movement on a four-by-four configuration. (**a**) 4 × 4 configuration. (**b**) illustrates the movement of the shuttles and rings. The shuttle and rings move in programable increments. It begins with the shuttle moving to the first ring to collect a droplet, the ring then rotates clockwise towards the next radii to collect the next droplet. These movements are performed until all the droplets are produced and the combinations dispensed.

With dilutions the following restriction is applied,

(7) $$N_{\mathrm{ring}}\;=\;N_{\mathrm{dil}}\;=\;r$$

then the required number of droplets is,

(8) Cr′n=r Nradr − 1 (Nrad − r + 1)

by inspection, which cannot be proven, only shown. All of _n_C_r_ and _n_C_r_^′^ are integers. The inequality, (6), becomes more numerically advantageous as the number of combinations increases, Table 1. In the simple example of a three by three configuration, Figure 4, the number of droplets needed is reduced from eighty-four to twenty-seven. The addition of four components, three drugs and a cell or multicell line, is shown in Figure 5 for a four radii configuration.

## 4. Reducing the Number of Combinations by Dilutions

Taking different dilutions of the drugs is important as it not only allows for a range of concentrations to be tested, but also reduces interference from other substances that may be present in the samples [32]. Therefore, dilutions are often a necessary part of any drug testing experiment. In this context, dilutions can also be used to reduce the combinatorial explosions. For a forty-eight-by-four system, this offers the user a maximum input of forty-eight different drugs, in dilutions of four. Using Equation (1), a total of some fifty-four million droplets need be produced to complete all possible combinations. Every droplet will need to be repeated several times, so the maximum number of droplets is over two-hundred million. Hence the need to reduce it.

**Figure 6:**
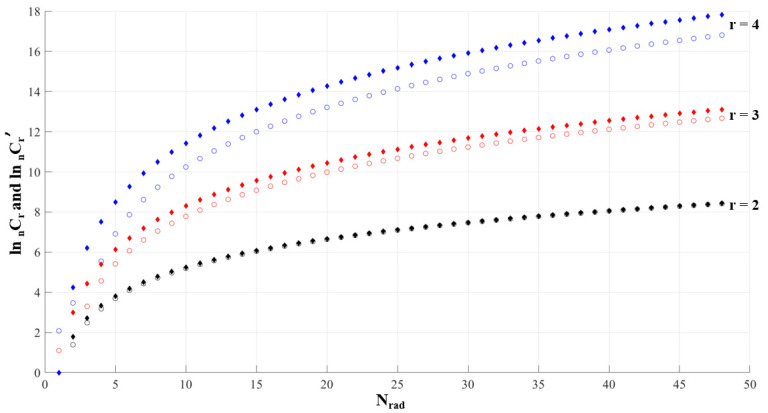
The logarithmic difference between Equation (1) and Equation (8). The circular points are _n_C_r_ and the diamonds _n_C_r_^′^. As r increases so does the difference between _n_C_r_ and _n_C_r_^′^.

With dilutions, many of the droplets contain redundant samples, combinations that contain two or more dilutions of the same drugs. These samples are not needed. Therefore, by taking account of dilutions, the number of total droplets to be produced for the test run can be significantly reduced, Equation (8). Figure 6 illustrates the reduction in droplets between _n_C_r_ and _n_C_r_^′^. For a forty-eight-by-four system, with *r* = 4, the number of droplets reduces from around fifty million to around nineteen million.

## 5. Commercial Instrument

**Figure 7:**
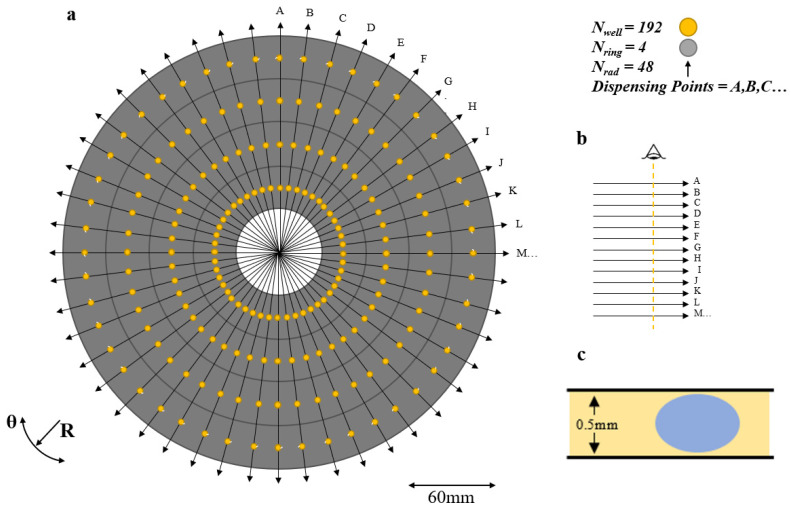
Proposed Commercial 48 × 4 Configuration. (**a**) 48 × 4 system containing forty-eight drugs in four different dilutions. Each radius of the system contains a single drug, with each ring in that radius containing a dilution of that drug. Each dot represents a well containing a drug dilution. Any number of radii and rings may be utilised depending on the needs of the user. At maximum some produces 53 million combinations of four. (**b**) After exiting the rotors, the droplets are read using either fiber optics or laser LEDs. (**c**) Typical droplet completely surrounded by silicone oil.

The proposed commercial design has a maximum of forty-eight drugs in combinations of four, Figure 7. With no dilutions, this has of order of fifty-five million droplets required and over a million rotor movements to dispense them. There are many lesser configurations, depending on which wells are filled and which algorithm subroutine is selected.

If the maximum is chosen with dilutions, there are then forty-eight different drugs and four dilutions of each drug. Equation (4) gives 19,906,560 combinations and 414,730 positions to dispense them. This requires of order forty-eight days plus the well refill time. In personalized medicine, this is a limitation on the use of multiple drug combinatorial discovery with organoids. For these reasons machine learning is proposed.

## 6. Machine Learning

Machine learning (ML) uses algorithms to process data, learns from it, and makes a prediction about the future state of any new data set [33]. With ML, a system can be trained to be able to predict the outcome of a task by using a large amount of data and algorithms. In the context of combinatorial drug screening, ML has the potential to reduce the scale by speeding up testing. This is particularly beneficial for personalised medicine as accurate predictions to tailor patient-specific treatments would be faster. ML can be used to focus in on a specific combination when synergy is detected using the Loewe additivity or Bliss model for example [18]. When a ML test is run, a circumferential movement on a single ring would be step one. If synergy were to be found between three drugs, then the system will focus on that region (the drugs in that area). This can be achieved through scoring. If the drugs display additive interactions, in which there is a difference in the way they interact together, they would be scored 1. If they display antagonistic interactions, in which they hinder the response, they would be scored 0, and if they show synergistic interactions, they score 2. This is one method of synergy-searching. After one circumference is tested with no synergy, step two would be to test another, etc. This may be a more hands-on process of filling only the wells in a circumference until synergy is found.

## 7. Conclusions

It is demonstrated how the automatic microfluidic design has great flexibility in the many configurations that may be chosen by the user. A 192 well system is proposed which can produce over 50 million combinations for synergy-searching. It is shown how this number is reduced by taking dilutions, by machine learning, and by more user interaction with the instrument. Throughput and drug volume consumed may follow a type of Moore’s law in the future.

## 8. Patents

Davies, M. Microfluidic device with channel plates. WO 2015/173651; EP3142790, 20 March 2019.

Davies, M; Galvin, M. Rotating mechanical device for generating a combinatorial droplet library. WO 2016/207721; EP3314046, 24 April 2019.

## Figures and Tables

**Table 1:** Different configurations for the proposed 48 × 4 commercial design. _n_C_r_ and _n_C_r_^′^ for different builds and combinations are given. The number of combinations ***r*** corresponds to the number of rings, ***N_ring_***. Each radius has a different drug and each ring a different dilution. The effect of dilution can be observed in this table. The effect of machine learning can also be seen by comparing the time taken for the droplets when dilutions are considered to the time taken with machine learning. All configurations are for the same instrument.

r	N_rad_	_n_C_r_	_n_C_r_^′^	N_c_^′^	N_r_^′^	N_p_^′^	Time (days)	Time ML (days), Single Ring, See ML Section
**2**	2	6	4	2	2	2	0.0002	0.000001
**2**	3	15	12	6	6	4	0.0004	0.000003
**2**	4	28	24	12	12	6	0.0007	0.000007
**2**	6	66	60	30	30	10	0.001	0.00002
**2**	8	120	112	56	56	14	0.002	0.00003
**2**	10	190	180	90	90	18	0.002	0.00005
**2**	20	780	760	380	380	38	0.004	0.0002
**2**	30	1770	1740	870	870	58	0.007	0.0005
**2**	48	4560	4512	2256	2256	94	0.01	0.0014
**3**	3	84	27	3	24	9	0.001	0.000002
**3**	4	220	96	12	84	24	0.003	0.000007
**3**	6	816	432	60	372	72	0.008	0.00004
**3**	8	2024	1152	168	984	144	0.02	0.0001
**3**	10	4060	2400	360	2040	240	0.03	0.0002
**3**	20	34,220	21,600	3420	18,180	1080	0.13	0.0021
**3**	30	117,480	75,600	12,180	63,420	2520	0.3	0.0073
**3**	40	280,840	182,400	29,640	152,760	4560	0.5	0.0179
**3**	48	487,344	317,952	51,888	266,064	6624	0.5	0.0313
**4**	4	1820	256	4	252	64	0.007	0.000002
**4**	6	10,626	2592	60	2532	432	0.05	0.00003
**4**	8	35,960	10,240	280	9960	1280	0.15	0.0002
**4**	10	91,390	28,000	840	27,160	2800	0.3	0.0005
**4**	20	1,581,580	544,000	19,380	524,620	27,200	3.15	0.0117
**4**	30	8,214,570	2,916,000	109,620	2,806,380	97,200	11.3	0.0661
**4**	35	15,329,615	5,488,000	209,440	5,278,560	156,800	18.1	0.1263
**4**	40	26,294,360	9,472,000	365,560	91,06440	236,800	27.4	0.2204
**4**	48	54,870,480	19,906,560	778,320	19,128,240	414,720	48	0.4692

## Nomenclature

**Symbol**	**Description**
N_c_	Number of circumferential movements
N_c′_	Number of circumferential movements with dilutions
N_dil_	Number of dilutions
N_r_	Number of radial movements
N_r′_	Number of radial movements with dilutions
N_rad_	Number of radii of the configuration
N_ring_	Number of rings of the configuration
N_p_	Number of positions the system must perform
N_well_	Number of wells
CnRm	Number of droplets formed radially
CnR′m	Number of droplets formed radially with dilutions
CnCm	Number of droplets formed circumferentially
CnC′m	Number of droplets formed circumferentially with dilutions
_n_C_r_	Number of droplets without dilutions
_n_C_r_^′^	Number of droplets with dilutions
n	Number of wells on the system
r	Number of combinations
z	Shuttle dispensing direction
R	Radial movements, radial direction
θ	Angular movements
